# AI in drug development: a multidisciplinary perspective

**DOI:** 10.1007/s11030-021-10266-8

**Published:** 2021-07-12

**Authors:** Víctor Gallego, Roi Naveiro, Carlos Roca, David Ríos Insua, Nuria E. Campillo

**Affiliations:** 1 Institute of Mathematical Sciences (ICMAT-CSIC), Nicolás Cabrera 13-15, 28049 Madrid, Spain; 2 AItenea Biotech S.L. Parque Científico de Madrid, Faraday, 7, 28049 Madrid, Spain; 3 ICMAT-CSIC and Dept. of Statistics and OR, U. Compl. Madrid, Madrid, Spain; 4 CIB-Margarita Salas (CSIC), Ramiro de Maeztu, 9, 28040 Madrid, Spain

**Keywords:** Drug development, Chemoinformatics, Artificial intelligence, Machine learning, Deep learning, Bayesian methods, Decision support

## Abstract

**Abstract:**

The introduction of a new drug to the commercial market follows a complex and long process that typically spans over several years and entails large monetary costs due to a high attrition rate. Because of this, there is an urgent need to improve this process using innovative technologies such as artificial intelligence (AI). Different AI tools are being applied to support all four steps of the drug development process (basic research for drug discovery; pre-clinical phase; clinical phase; and postmarketing). Some of the main tasks where AI has proven useful include identifying molecular targets, searching for hit and lead compounds, synthesising drug-like compounds and predicting ADME-Tox. This review, on the one hand, brings in a mathematical vision of some of the key AI methods used in drug development closer to medicinal chemists and, on the other hand, brings the drug development process and the use of different models closer to mathematicians. Emphasis is placed on two aspects not mentioned in similar surveys, namely, Bayesian approaches and their applications to molecular modelling and the eventual final use of the methods to actually support decisions.

**Graphic abstract:**

Promoting a perfect synergy 
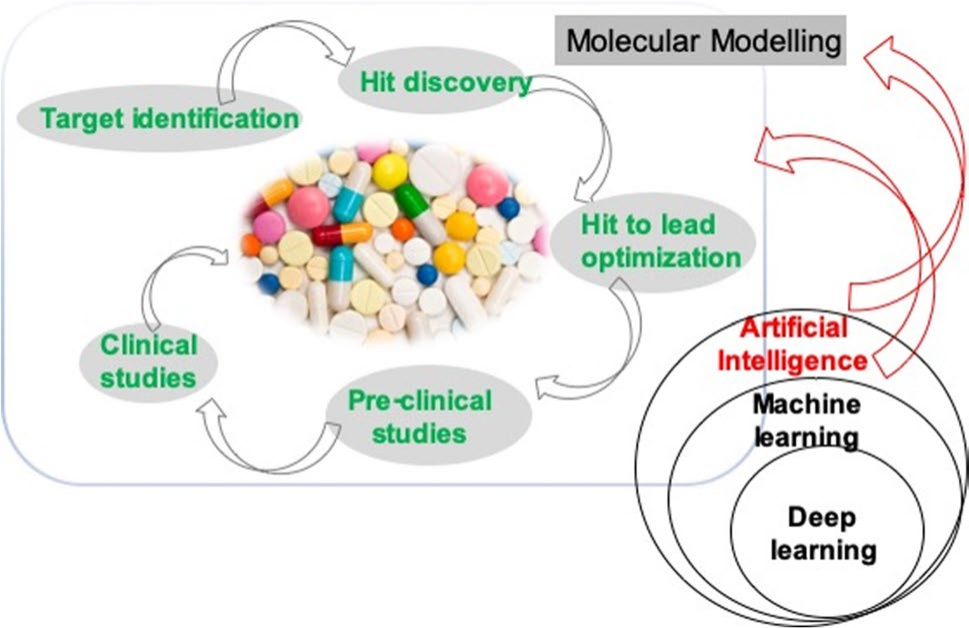

## Introduction

The concept *artificial intelligence* (AI) was first described by McCarthy in 1956 as “the science and engineering of making intelligent machines” although six years before, Turing had forwarded the idea of using computers to emulate human behaviour and intelligence [1]. Since then, and after several waves of popularity, the development of AI, and specially its more statistical branch known as machine learning (ML), has reached spectacular successes in many applied domains, in part due to the popularity of deep learning (DL) (see, e.g. [2, 3] for relevant reviews). In the healthcare sector, AI methods and tools have been applied both to the so-called virtual and physical areas, the latter one referring to the development of medical physical devices and objects [4]. Our focus will be more on the virtual area, specifically in the application of AI to the drug development process. Such process is typically very lengthy and complex, with several stages from the disease and therapeutic target identification until a drug reaches the market, and entails large monetary costs and a high attrition rate [5].

**Fig. 1:**
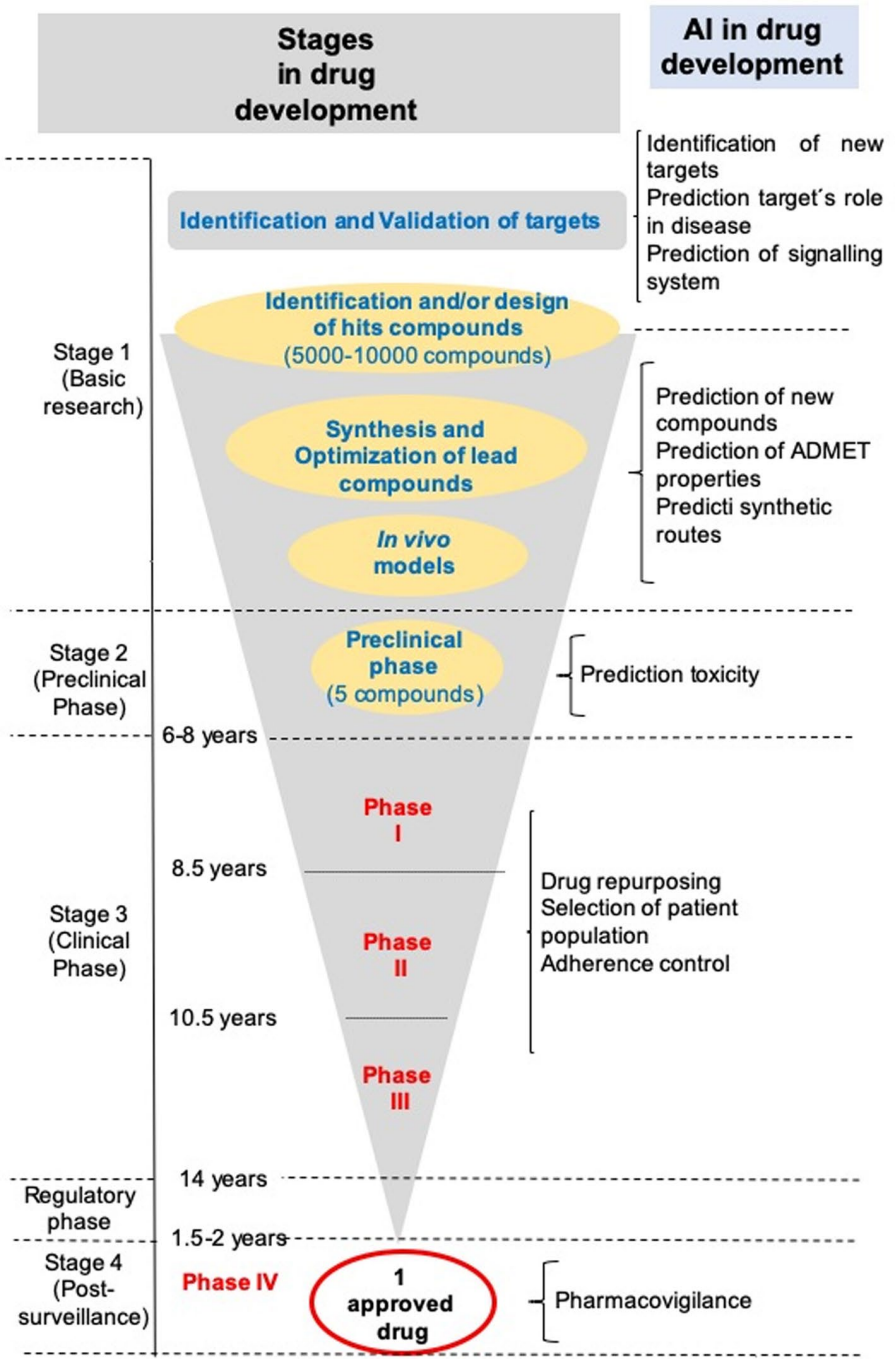
Drug development process showing the application of AI at each stage. Adapted from [6, 7]

Indeed, drug discovery and development can be viewed as a pipeline with four major stages (Fig. 1) which to a large extent, it actually becomes somewhat of a steeplechase in which many competing molecules start but very few reach the finish. As an example, between 2002 and 2012, the failure rate in developing new drugs for the treatment of Alzheimer’s disease reached 99.6%. Moreover, approximately only 38% of new chemical entities in Phase IIb and Phase III clinical trials reached the market, being the major sources of attrition failures in safety and efficacy followed by those in relation to formulation, pharmacokinetic and bioavailability [6].

Over the last decades several computational methods have been introduced to reduce drug discovery times and costs, as well as improve the development process quality and its success rate [8]. However, there is still much work to be done to streamline this process through the use of innovative technologies, including those from the domain of AI. Indeed, different AI tools are being applied in all steps of the drug development process, including the identification and validation of molecular targets, finding and optimising hit and lead compounds, the synthesis of drug-like compounds, the prediction of ADME-Tox, and, even, clinical trials (Fig. 1), see [6, 9–12] for reviews.

In particular, Schneider et al. recently showcased the benefits of using AI tools in drug development due to the potential of predictive models for navigating large chemical databases [10]. However, several related challenges remain, including: (i) the availability of robust and appropriate datasets; (ii) a *de novo* design for exploring the chemical space; (iii) the use of multi-objective optimisation to simultaneously pursue several drug-like properties; (iv) the reduction of cycle times; (v) a real synergy between mathematicians and medicinal chemists (MC); and (vi) the generation of synergies between AI and computational chemoinformatics methods.

This review aims at addressing several of such issues, by, on the one hand, bringing a mathematical vision of some key AI methods useful in drug development closer to MC (section "Basic machine learning methods for drug development") and, on the other hand, by presenting the drug development process and the use of different models closer to the mathematical community (section "Applications in molecular modelling"). Our focus is on the first stage of the drug development process, but sees our final discussion for applications in the other stages.

## Basic machine learning methods for drug development

This section provides a brief overview of ML methods for drug development, later illustrated in Sect. 3. We emphasise several aspects not frequently mentioned in other surveys. First of all, for MC, the proposed models are of relevance mainly because they serve to support the complex decision-making processes entailed in their activities. As a consequence, we emphasise Bayesian approaches to such models, because they provide improved uncertainty estimates in the predictions, this being crucial in decision support; they have enhanced generalisation and improved model calibration capabilities; and, finally, they allow us to model prior medical and chemical expert information both from the application domain and also through the use of sparsity-inducing priors. All this leads to improvements in learning.

We first focus on methods prior to deep learning (sections "Supervised learning" and "Classification") and then emphasise deep learning models (section "Deep learning") and reinforcement learning (section "Reinforcement learning").

### Supervised learning

We provide a brief explanation of supervised learning methods for the MC toolkit. Detailed descriptions may be seen in, e.g. [13].

#### Classification

**Fig. 2:**
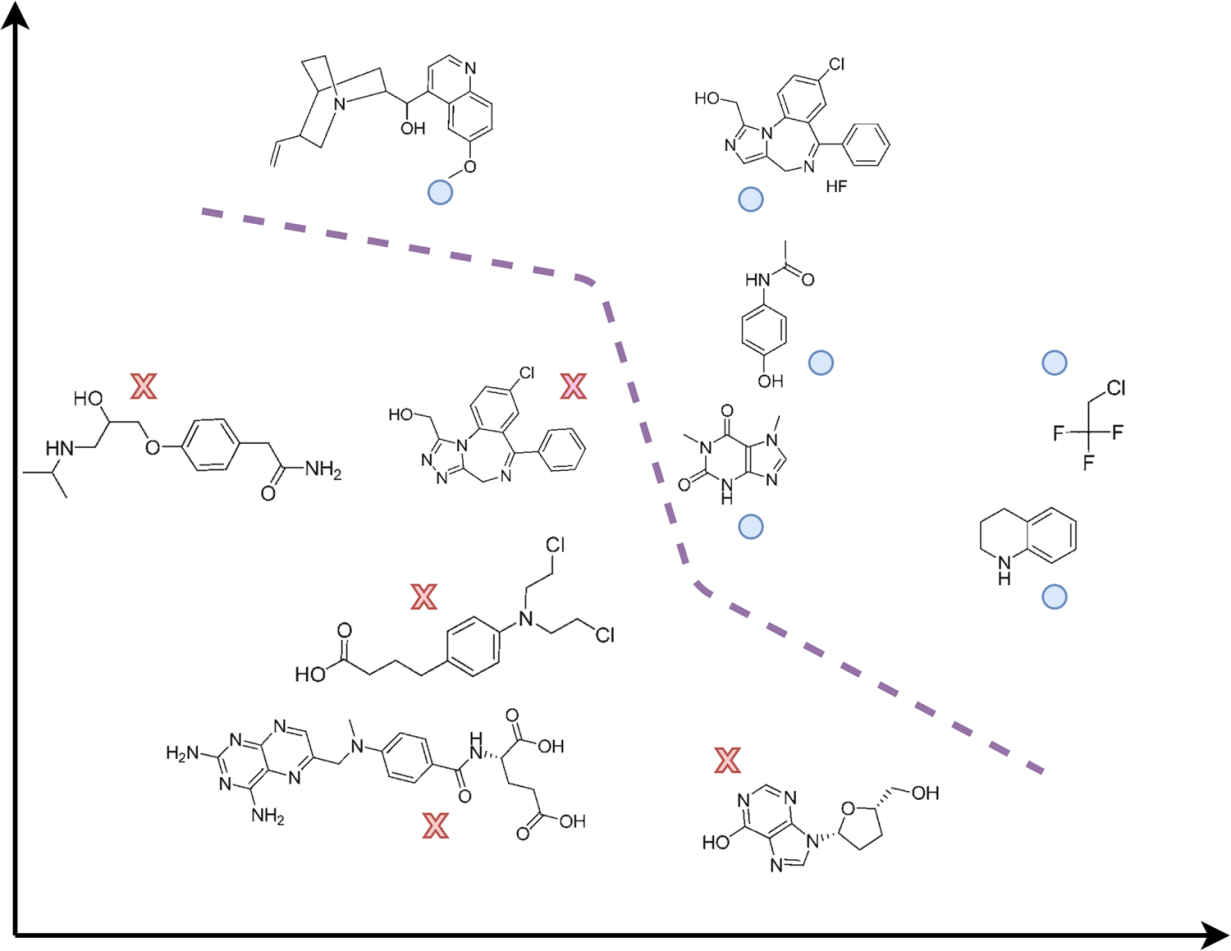
An schematic view of classification into two classes

In classification settings, a decision maker (DM) (the MC or, better, a computer system delegated with such task) receives instances that belong to one of *K* possible classes denoted $y \in \lbrace y_1, \dots , y_K \rbrace$. Instances have *p* covariates *x* whose distribution informs the DM about their class *y*. As an example, when modelling a quantitative structure–activity relationship (QSAR), instances refer to molecules, the response variable *y* could refer to a categorical measure of biological activity, and covariates consist of different molecular descriptors or measures of relevant properties of the molecules. The goal of classification is to obtain accurate predictions about the class of a new molecule with covariates *x* and is typically split into two phases referring to inference (training) and decision making (operations). Figure 2 depicts an schematic view of classification, in which eleven molecules have been classified into two classes: those that permeate the Blood-Brain Barrier (denoted with black circles) and those that do not (denoted with red crosses). Note that the model has learnt a decision surface (denoted by the purple dashed lines), used to classify new molecules.

At the training stage, a distribution *p*(*y*|*x*) predicting the instance class *y* given features *x* is learnt. For this, we may adopt a *generative* approach: based on training data, models *p*(*x*|*y*) and *p*(*y*) are learnt and *p*(*y*|*x*) is deduced via Bayes formula; a popular example in medicinal chemistry is Naive Bayes [14]. Alternatively, we may adopt a *discriminative approach* to directly learn *p*(*y*|*x*) from data; major examples include logistic regression [15] and feed-forward neural networks (NN), see section "Deep learning" for some details.

For learning the relevant models from data, we could adopt a frequentist approach in the training stage. Based on training data ${\mathcal {D}}$, a regularised maximum likelihood estimate ${\hat{\beta }}$ is obtained and plugged into the classification model. In large-scale models with high-dimensional data, like deep NNs, training is usually implemented through *stochastic gradient descent* (SGD) [16]. Alternatively, we could adopt a Bayesian approach modelling the MC expert beliefs as well as the structural information available about the incumbent parameters through a prior distribution; use it to assess the posterior, given the experimental data; and, finally, estimate the predictive distribution for classification purposes. Note that this distribution quantifies, in a formal manner, the remaining uncertainty about the class of the molecule described by covariates *x* after having observed the training data.

The second stage entails making a *decision* about the class of a newly observed instance. For this, the DM uses the learnt predictive model *p*(*y*|*x*) and the utility $u (y_c, y_i)$ to be attained when the actual class is $y_i$ and the suggested one is $y_c$ given the observed instance *x*. Following decision-theoretical principles [17], the DM searches for the class maximising expected utility solving

1 $$\begin{aligned} \arg \max _{y_c} \sum _{i=1}^K u (y_c , y_i ) p (y_i | x ). \end{aligned}$$

Decision-making aspects have been largely ignored in drug development. The common approach is to assign instance *x* to the class with maximum predictive probability $p(y \vert x)$. This is equivalent to using a 0-1 utility function $u (y_c, y_i) = {\mathbb {I}}(y_c = y_i)$, where ${\mathbb {I}}$ is the indicator function, which implicitly gives the same importance to every misclassification error. However, this is not the case in many applications during the identification/design of ligand compounds or the optimisation of hit/lead compounds. Indeed, such decision is key in determining what compounds will be synthesised or further studied. As an example in a MC program to develop inhibitors of BACE-1, it is necessary to assess whether the effort (time/money) of synthesising and studying a moderate inhibitor is worth it. In general, the utility is characterised as a matrix whose entries assess the utility that the classifier perceives when she declares an instance of class $y_i$ when its actual label is $y_j$. For this, it aggregates multiple objectives balancing the importance of different misclassification errors. Moreover, ideally it should integrate the DM’s risk attitudes.

#### Regression

In regression settings, the response variable *y* is continuous. As before the *p* covariates *x* informs about *y*. For instance in QSAR models, biological activity is often measured as the level of concentration of a substance required to induce a certain response: *y* would be this concentration measure, and the covariates could be, as before, different molecular descriptors.

As with classification, regression problems can be broken down into inference (training) and decision (operational) stages. The first stage uses training data to learn about the distribution of *p*(*y*|*x*). Typically, parametric models $p(y|x, \beta )$ specified through $y = f_{\beta } (x) + \epsilon$ are used. Here, $\epsilon$ is generally a zero-mean Gaussian noise. Note, for example, that if $f_{\beta } (x) = \beta ' x$, we recover linear regression models.

Frequentist approaches entail finding, possibly regularised, maximum likelihood estimates ${\hat{\beta }}$ for the parameters $\beta$, and using the plug-in model $p(y \vert x, {\hat{\beta }} )$ for prediction purposes. Bayesian approximations focus on estimating the parameters given the observed data ${\mathcal {D}}$ through the posterior distribution $p(\beta \vert {\mathcal {D}})$; for prediction, the posterior predictive distribution $p(y \vert x, {\mathcal {D}} )$ of a newly observed instance with features *x* is used. Some important classical regression approaches in MC include boosting, NNs, *k* nearest neighbours, random forest (RF), relevance vector machines, partial least squares, and support vector regression (SVR), see, e.g. [18]. Most of them have their Bayesian counterpart.

As in classification, the *operational* stage utilises *p*(*y*|*x*) to make a forecast of the biological activity of interest for a new observed instance. As before, if *u*(*z*, *y*) is the utility perceived for deciding *z* when the actual value is *y*, the optimal forecast is $\arg \max _{z} \int u (z , y ) p (y | x ) dy$. As an example, if $u (z , y ) = (z-y)^2$, the optimal point forecast is the expected value of the predictive distribution.

Often, the interest in drug development is to find molecules that maximise biological activity. Usually, we have just a few molecules for which we know this measure, and the goal is to sequentially synthesise more molecules, in order to get one that is good enough for our purposes (e.g. has good ADME-Tox properties). However, synthesising new molecules is expensive and thus, we need a procedure to guide this search. This is the goal of Bayesian optimisation, [19]: first, a Bayesian regression model is fitted for the data, usually based on a Gaussian Process (GP). As before, each molecule is described by a feature vector *x*. Given an unseen molecule *x*, the predictive distribution *p*(*y*|*x*) can be computed. Next, the utility associated with measuring this new molecule is assessed. A classical example is $u(x) = \max (0, y - y^*)$, where *y* is the biological activity of the candidate molecule and $y^*$ is the activity of the best molecule so far found. With this and the predictive distribution, the expected utility of any candidate molecule can be determined, and the goal would be to iteratively search for molecules maximising this expected utility, and update the regression model with the discovered molecules, until resources are exhausted.

### Unsupervised learning

Whereas supervised learning methods are used to predict future values of data categories, unsupervised learning methods are used mainly for exploratory purposes. In drug development, it is undertaken with two main aims: dimensionality reduction (e.g. to facilitate visualisation of high-dimensional data) and clustering (e.g. to identify similar molecules according to their representation).

*Dimensionality reduction* These techniques seek for meaningful low-dimensional representations of high-dimensional feature vectors. As an example, when working with molecules represented by a high number of molecular descriptors, projecting these into a low-dimensional space is useful for visualisation purposes.

As with supervised learning, both Bayesian and frequentist unsupervised techniques exist. Among frequentist approaches, we find linear and nonlinear methods. Linear methods seek for a linear projection of the data into a low-dimensional space, being principal component analysis (PCA) the most well-known [20]. Similarly, nonlinear methods find nonlinear projections; some of the most commonly used are t-distributed stochastic neighbour embeddings (tSNE) [21], autoencoders (AE) [22] and the uniform manifold approximation and projection (UMAP) method [23].

The Bayesian approach entails assuming a generative model for the observed data that depends on some low-dimensional parameters, usually referred to as latent variables. All relevant inferential information about the low-dimensional representation (the latent variables) is thus encoded in their posterior distribution given the observed data. Some important Bayesian approaches for dimensionality reduction include probabilistic PCA [24], GP latent variable models [25] and variational autoencoders (VAE) [26].

**Fig. 3:**
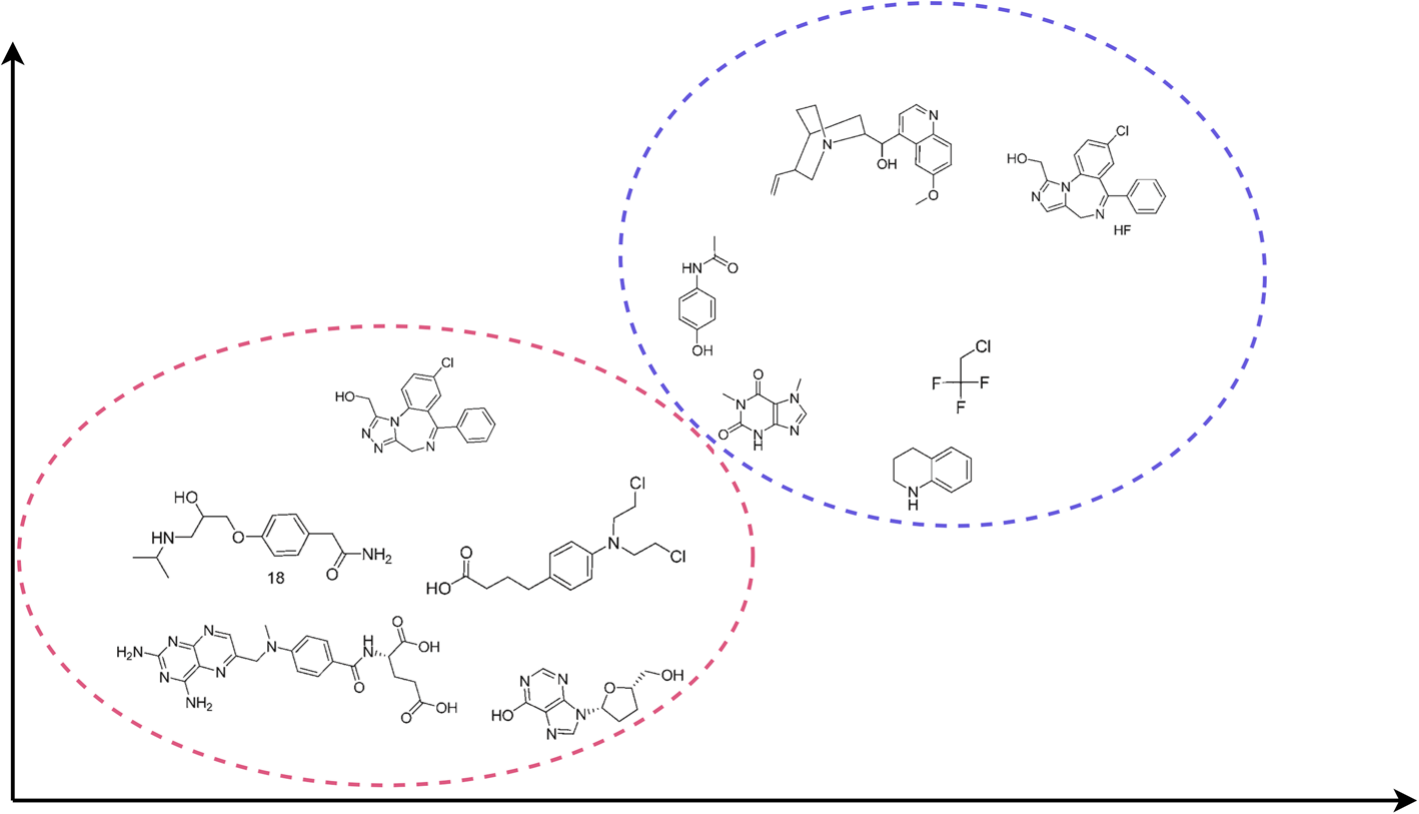
An schematic view of clustering

*Clustering* These techniques aim at identifying relevant groups among the instances, so that those within a same cluster are more similar than those belonging to different ones. Figure 3 depicts an schematic view of clustering, using the same sample molecules from section "Supervised learning".

Several classical approaches for clustering exist. One the most widely used is *k*-means [27], seeking for *k* cluster centres and assigning each data point to a centre, so as to minimise the total within-cluster sum of distances. Hierarchical clustering [27] techniques are another important group of cluster analysis methods. While the previous methods require fixing the number of clusters to be found at the beginning, hierarchical ones produce a hierarchy which may be cut at different depths to provide different numbers of clusters.

For all previous methods, we find their Bayesian counterparts. Among others, Gaussian mixture models [18] are a probabilistic generalisation of *k*-means. They are probabilistic models that assume that the data have been generated from a finite mixture of Gaussian distribution. In these models, inference about the mixture component weights and parameters is made in a Bayesian way. Bayesian hierarchical clustering has also been proposed in e.g. [28].

### Deep learning

Because of its importance in recent ML developments, we turn our attention to NN-based models, distinguishing different architectures that have made a significant impact in the field and are relevant in drug development.

#### Shallow neural networks

These approximate an *r*-dimensional response *y* based on *p* explanatory variables *x* through a model $y = \sum _{j=1}^m \beta _j \psi (x' \gamma _j) + \epsilon , \epsilon \sim N(0,\sigma ^2),$ where, originally, $\psi (\eta ) = \exp (\eta )/(1+\exp (\eta ))$. This is designated a NN with one hidden layer containing *m* hidden neurons and logistic activation functions $\psi$. As an example, the variable $y \in {\mathbb {R}}$ could refer to the continuous level of any property of interest, such as toxicity or solubility, and *x* could be a vector molecular descriptors.

Given *n* observations $D=\{ (x_i, y_i), i=1,...,n \}$, maximum likelihood estimation computes the log-likelihood and maximises it leading to the classical nonlinear least squares problem $\min _{\beta , \gamma } f (\beta , \gamma ) = \sum _{i=1}^n \left( y_i - \sum _{j=1}^m \beta _j \psi (x_i'\gamma _j) \right) ^2$. Quite early, researchers paid attention to the introduction of regularisers, such as weight decay $\ell _2$ penalisation [29], improving model generalisation through solving the optimisation problem $\min g(\beta ,\gamma ) = f (\beta ,\gamma ) + h (\beta ,\gamma ),$ where $h(\beta , \gamma )$ represents the regularisation term. Typically such problems are solved via steepest gradient descent [30], with gradients estimated via backpropagation, e.g. [31]. Moreover, similar models may be used for classification purposes, although this requires modifying the likelihood to, e.g.

$$\begin{aligned} p(y | x, \beta , \gamma ) = Multin(n=1, p_1 (x, \beta , \gamma ) , \ldots , p_K (x, \beta , \gamma ) ). \end{aligned}$$

Then, class probabilities are assessed through

$$\begin{aligned} p_k = \frac{\exp {\beta _k \psi (x'\gamma _k)}}{\exp {\sum _{k=1}^K \beta _k \psi (x'\gamma _k)}}. \end{aligned}$$

We can also formulate the Bayesian counterpart of a shallow NN, by introducing an informative prior probability model which is meaningful as parameters are interpretable, see [32] for details, who introduce efficient Markov chain Monte Carlo (MCMC) schemes for inference and NN architecture selection. In particular, Bayesian approaches might come in very handy for drug development tasks, as, besides providing a point prediction, they supply the entire predictive distribution *p*(*y*|*x*), better informing decision making.

#### Deep neural networks

**Fig. 4:**
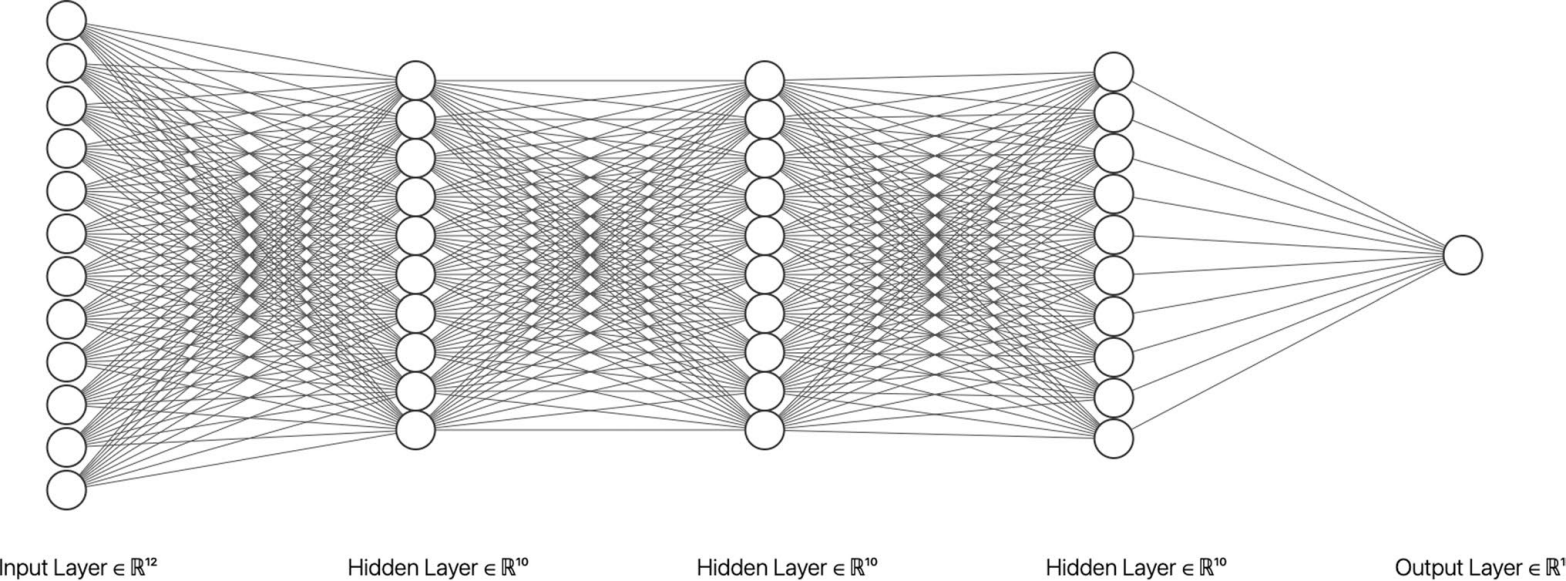
A deep NN architecture with three hidden layers

Training by backpropagation has been in use for many years by now. The decade of the 2010’s saw major developments leading to the current boom around deep learning (DL). This refers to inference and prediction with deep NNs (DNNs) which may be defined through a sequence of functions $\lbrace f_0, f_1, ..., f_{L-1} \rbrace$, each parameterised by some weights $\gamma _l$ of dimension $m_l$ (the corresponding number of hidden nodes) with the output of each layer being the input of the following one, as in $z_{l+1} = f_l ( z_l, \gamma _l)$. Lastly, a prediction from the hidden activations of the last layer is computed as before through $y = \sum _{j=1}^{m_L} \beta _j z_{L,j} + \epsilon , \epsilon \sim N(0,\sigma ^2)$. An example of the architecture of a deep neural network with three hidden layers is shown in Fig. 4.

Modern architectures do not longer require the $f_l$ functions to be sigmoidal, like the logistic functions above, and include the rectified linear unit (ReLU), the leaky ReLU or the exponential LU. In particular, these functions mitigate the vanishing gradient problem [33] that plagued earlier attempts with deep architectures using sigmoidal activation functions.

DNNs have important advantages over most other ML methods, since they can straightforwardly model several activities at a time (multi-task models), may generate novel chemical features and enable inverting a QSAR model, i.e. designing molecules directly from the model (through generative models). Note, though, that DNNs also have undesirable characteristics like requiring more tuning of training parameters, being more demanding computationally, taking longer to predict, and defying interpretation oftentimes.

Beyond the above generic deep architectures, a few important specialised models have emerged which are relevant in certain MC applications.

*Convolutional neural networks* CNNs were originally designed to tackle vision tasks and related signal processing applications. Stemming from the work by Le Cun and coauthors [34, 35] and their original LeNet5 design, they achieved major successes in competitions [36] leading to architectures like AlexNet [37], VGGNet [38] or GoogleNet [39], reaching superhuman performance in image recognition tasks. In CNNs, the layer transformation is taken to be a convolution with some 2D or 3D kernel; this makes the network able to recognise patterns independently of their location or scale in the input, a desirable property in computer vision tasks known as spatial equivariance, which improves generalisation capabilities. In the case of drug discovery, molecules can be described as a graph. Related to CNNs are graph NNs [40], which instead of receiving a grid of points such as an image, receive as input a more general graph (a set of atoms and the connections between them).

*Recurrent neural networks* The original domain for RNNs was sequence processing, as in natural language processing (NLP) [41, 42]. They have feedback connections which make the network aware of temporal dependencies in the input. The classical example is the Elman network [43]. Backpropagating through long sequences may lead to problems of either vanishing or exploding gradients [44]. As a consequence, gating architectures improving the stability have been proposed, and successfully applied in real-life tasks, including gated recurrent unit (GRU) networks [45] and long short-term memory (LSTM) networks [41]. In chemical design tasks, the most popular case is to treat the SMILES representation of the molecule as the input sequence to a RNN to provide novel molecular descriptions.

*Transformers* These architectures substitute the sequential processing from RNNs by a more efficient, parallel approach inspired in attention mechanisms [46, 47]. Their basic building components are scaled dot-product attention layers that produce activations for every element in the sequence. Each layer of a transformer model usually comprises several parallel layers, enabling the net to pay attention to different parts of the input simultaneously. Attention layers are alternated with feed-forward ones in what is designated an encoder block. These can be stacked until a final layer outputting classification probabilities. If the task requires producing outputs that are variable in length, as in automatic translation or summarisation, decoder layers must be used, which replicate the work of encoders until output generation. Since transformer-based models are more amenable to parallelisation, they have been trained over massive datasets in the NLP domain, leading to architectures such as Bidirectional Encoder Representations for Transformers (BERT) [48] or the series of Generative pre-trained Transformer (GPT) models, e.g. [49]. Regarding molecular representations, ChemBERTa has been recently introduced leading to encouraging results on MoleculeNet tasks [50, 51]. Transformers have also shown excellent results at protein structure design [52].

*Generative models* The models from the previous paragraph belong to the discriminative family of models, which directly learn the conditional distribution *p*(*y*|*x*) from the data. Alternatively, generative models take a training set, consisting of samples from a distribution $p_{data}(x)$, and learn to represent an estimation of that distribution, resulting in another probability distribution $p_{model}(x)$. Then, one could fit a distribution to the data by performing maximum likelihood estimation, or maximum a posteriori estimation if a prior over the parameters $\theta$ are also placed. An important family of generative models are called autoencoders [53]. They perform dimensionality reduction using a sequence of nonlinear transformations [22] followed by a reconstructing model where the goal is to learn how to generate from the reduced space a representation as close as possible to its original input. They can be regarded as a nonlinear extension of PCA. Of relevant interest are their probabilistic counterparts, variational autoencoders (VAEs) [54]. However, these models do not have a tractable density function, and one must resort to approximation techniques.

In [83], the authors propose a VAE architecture to transform the discrete molecular representation into a continuous latent space and then perform optimisation to search for properties of interest. Since then, a large number of variations of VAE-like models have been developed, such as the GrammarVAE [55] or the Constrained Graph VAE [56].

*Generative adversarial networks* GANs perform density estimation in high-dimensional spaces formulating a game between a generator and a discriminator, parameterised as NNs, [57]. They belong to the family of generative models but do not explicitly model a distribution $p_{model}$, only generating samples from it. Each network has its own objective function, with both networks playing a minimax game. While GANs have already produced astonishing results in areas such as image generation [58, 59], they are still pervaded by problems such as training instabilities or mode collapse, in which the generator gets stuck on a mode and the samples generated lack diversity. For a comprehensive survey on the use of GANs in drug design and discovery tasks, see [60].

#### Computational issues

In principle, we could think of applying the optimisation approaches in section "Shallow neural networks" to DNNs. However, large-scale problems bring in two major computational issues: first, the evaluation of the gradient requires going through all observations becoming too expensive with large data sets; second, estimation of the gradient component for each point requires a much longer backpropagation recursion through the various levels of the deep network, entailing again a very high computational expense.

Fortunately, these computational demands are mitigated through the use of classical SGD methods [61] to perform the estimation [62]. SGD is the current workhorse of large-scale optimisation and allows training deep NNs over large datasets by mini-batching: rather than going through the whole data batch at each stage of gradient descent, just pick a small sample (mini batch) of observations and do the corresponding gradient estimation by backpropagation. Recent work has explored ways to speed up convergence, leading to SGD variants such as AdaGrad, Adadelta or Adam [63].

MCMC algorithms, mentioned in section "Shallow neural networks", have become standard in Bayesian inference [17]. However, they entail a significant computational burden in large datasets: computing the corresponding acceptance probabilities demands iterating over the whole dataset, which often does not even fit into memory. Thus, they do not scale well in big data settings. As a consequence, two major approximations have been proposed. The first one is stochastic-gradient Markov Chain Monte Carlo (SG-MCMC) methods, which use an estimate of the gradient plus some adequately sampled noise to explore the posterior distribution [64–66]. On the other hand, variational Bayes approaches approximate the posterior distribution with a simpler, tractable distribution, such as a Gaussian, by solving an optimisation problem to get the best approximation [67–69].

### Reinforcement learning

**Fig. 5:**
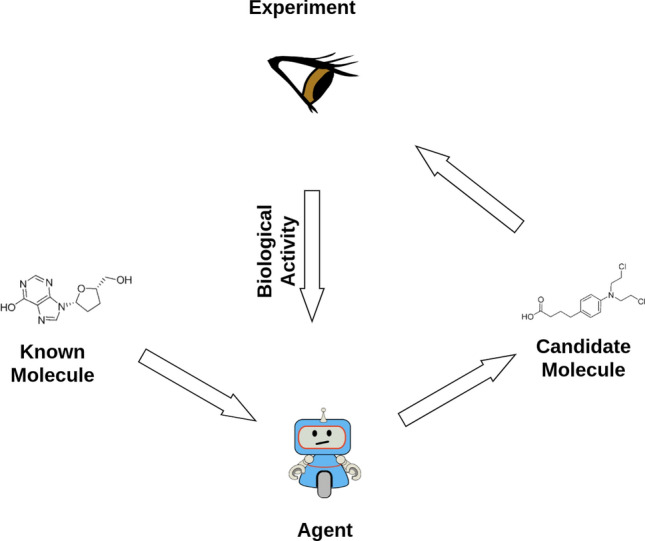
An schematic view of RL

Reinforcement learning (RL) [70], as opposed to supervised learning and similarly to unsupervised learning, does not require labelled data. Instead, an agent (driven by a RL model) takes actions sequentially while accumulating rewards from them. Its aim is learning a policy allowing an agent to maximise his total expected reward. As an example, assume for a moment that we have access to a predictive model (or an experiment) that, given a molecule, can predict a chemical property of interest, for instance biological activity, and give that molecule a score (the reward). Then, the RL agent consists of a generative model that as an action generates a molecule (this can be its SMILE code, a graph or any other representation of interest). This molecule is evaluated through the predictive model, receiving a reward, and is given to the generator as a feedback signal. We can iterate this loop many times, resulting in a generator that learns to produce molecules with a given chemical property (measured by achieving optimal reward). Figure 5 depicts an schematic view of this process.

The predictive component can be a black-box model provided by chemical software, but can also be obtained using the methods in section "Supervised learning". In that case, the generator is typically optimised through methods from one of two RL families based on either Q-learning [71, 72] or policy gradients [73, 74], which directly improve the agent’s policy. In the previous example, the policy could be based on a generator model from section "Deep learning", such as a VAE or a GAN. However, the predictive model could also be refined during the RL loop, as with the actor-critic family of algorithms [75].

## Applications in molecular modelling

As mentioned in Introduction, AI has a large number of applications and a growing importance in drug development processes. Conventional computational techniques helped in the last decades to generate great advances. However, the application of AI has entailed a major disruption in the search of solutions to chemical and biological problems. We sketch here some of the most commonly used conventional fields and techniques in drug development and how new AI applications are revolutionising the field.

### Molecular representation

To start with, let us highlight the importance of how the data are used, and the nature of the data as a fundamental part of the process. There are different representations to describe the structure and nature of chemical molecules, called molecular representations. The most important ones are based on *molecular graph representations* (mainly the Simplified Molecular Input Line Entry System, SMILES [76], and the *international chemical* identifier, InChI [77]); on *molecular descriptors* (the information in the molecular structure is encoded into one or more numbers capturing the structural characteristics and properties of the chemicals [78–80]); and on *molecular fingerprints* (bitstring fingerprints, of which each bit represents the presence (1) or absence (0) of a characteristic feature or molecular substructure [81, 82]).

Interestingly, recent powerful AI-based approaches have emerged like ChemBERTa, which adapts ideas from transfer learning and NLP using a BERT-like Transformer to improve the learned features of molecules, acting as refined fingerprints [51].

### *De novo* design

The generation of new chemical structures is one of the most promising applications of AI in drug development. In classic processes, MCs propose new compounds or substitutions to improve affinity or some particular property, always based on their knowledge and experience. However, the complexity of diseases and the uncertainty entailed by choosing the best therapeutic intervention points out that decision support in medicinal chemistry would greatly benefit from systematic approaches to drug design and discovery. *De novo* design aims at developing algorithms that facilitate this process through selection of novel molecules with optimised property profiles.

The development of new methodologies for *de novo* design is critical to delivering attractive ideas to MCs to make better decisions based on their experience. The situation has evolved so much in the last years, allowing *drug designer robots* to be already among us, actively participating in drug development at an industrial level. More in detail, there has been in the last decade important work concerning innovative methodologies for *de novo* design, applying VAEs, RNNs and other architectures (mostly Convolutional and Graph Networks).

*VAEs.* One of the pioneers in proposing novel VAE based methods to generate chemical structures was [83]. This methodology is based on a three-step process with an autoencoder, a multi-layer perceptron (MLP)-based predictor and a decoder, being capable of converting discrete SMILE strings into continuous vectors in a latent space, predicting new vectors with specific molecular properties and reconverting these vectors back into discrete SMILE strings. This methodology allows a gradient-based search through the chemical space with the ability of reconstructing organic molecules, capturing specific molecular characteristics of a training set, enabling *de novo* drug discovery with a particular type of properties. However, it was seen that some undesirable (as, for example, hardly synthesizable) molecules were also generated.

In order to solve the issue of creating valid structures, an adversarial autoencoder combining the properties of a molecule generator and a molecule discriminator was developed in [84]. This autoencoder was tested on a dataset of molecules with different tumour growth inhibition activities. The autoencoder creates fingerprints of molecules with the desired properties (anticancer properties in this case). Subsequently, [85] proposed an improved architecture called druGAN, which uses the VAE as a molecular descriptor generator combined with a GAN capable of generating novel chemical structures. druGAN showed improvement in feature extraction, generation capacity and error reconstruction, showing potential for its application in drug design. This also led to the use of VAE to generate new molecules against dopamine type 2 receptor with predicted activity [86].

*RNNs*. RNNs have been used as well to generate new chemical entities *de novo* [87, 88], specifically generating focused molecule libraries through transfer and RL in SMILES. The RNN is able to learn the probability distribution of characters in a SMILES string, writing structurally valid SMILES. Thus, it can be considered a generative model for specific and novel molecular structures. The model pre-trained on a public dataset of molecules was tuned on a small set of target-specific active compounds, being able to create new structures with desired activity against *Staphylococcus aureus* and *Plasmodium falciparum* [88].

A similar RNN model was also developed for *de novo* drug design and, for the first time, in molecular design in fragment growing [89]. This method uses generative RNN containing long short-term memory (LSTM) cells, capturing the syntax of molecular representation in terms of SMILES strings and learning pattern probabilities used for *de novo* SMILES generation. Additionally, the RNN’s predictions can be fine-tuned for specific molecular targets by employing transfer learning. Merk [90] developed in 2018 a deep RNN model with LSTM cells for *de novo* ligand generation. This approach requires only a small set of known bioactive template structures to capture relevant structural features for the target of interest. The focus was on the design of novel Retinoid-X-Receptor modulators.

Several additional examples of recent work in this field are based on Deep Q-learning, a RNN able to look for molecules with specific molecular properties such as cLogP and QED drug-likeness [91]; a policy-based RL approach to adjust RNNs to produce molecules with specific user-defined properties [92]; RL for Structural Evolution, an actor-critic RL approach in which the generator network is a RNN [93]; and ORGAN, a framework to optimise an arbitrary object in a sequence generation task, applying RL to control properties, such as drug-likeness, solubility and synthesizability of the generated samples [94].

Another recent exciting application of RNNs is DESMILES [95], a DL model that generates a small molecules set chemically related to a given ligand, using molecular fingerprints and translating it to a sequence of SMILES strings to estimate the probability of matching the fingerprint. After training, the RNN was fine-tuned using datasets to improve the biological activity against the D2 Dopamine Receptor, drug-likeness (QED) and logP.

*Other architectures.* Of special mention is the directed-message passing deep NN model [96], which converts the molecule representation into a continuous vector via a directed bond-based message-passing approach [97]. Similar models have been applied to antibiotic discovery [98]. A graph attention mechanism was adopted by the model for drug discovery [99].

### 3D-pharmacophore models

A pharmacophore is the 3D alignment of features that are necessary for the binding of compounds to a pharmacological target. 3D-molecular similarity methods are very useful and powerful tools to identify lead compounds in ligand based-virtual screening based on the principle that similar compounds would have similar bioactivity. A pharmacophore can be derived either in a structure-based way using the complementarities between a compound and its binding site, or in a ligand-based way, through structural alignment of a set of active ligands (in the bioactive conformation) and identifying the important common chemical geometry features [100]. However, these methods still present important limitations: (i) bioactive conformations of the structures are usually not known and (ii) dependence on structurally similar molecules [101]. Pharmacophore features derived from the 3D-structural alignment are being used for the development of new ML methods to improve both prediction of binding sites and the ranking of docking poses. Ballirani et al. developed a knowledge-based approach (HS-Pharm) employing atom-based fingerprints of known ligand-binding pockets as input to a RF model allowing a ranking of cavity atoms that should be targeted for ligand binding [102]. Sato et al developed a pharmacophore-based interaction fingerprint (Pharm-IF) using machine learning techniques such as SVMs and RFs instead of similarity-based ranking to improve docking pose ranking [103]. A CNN was developed to detect cavities and predict binding affinities, employing pharmacophoric descriptors to train the model [104].

### Quantitative structure–activity relationship (QSAR) models

QSAR modelling is a widely applied computational approach that relies on the premise that structurally similar molecules have similar physicochemical and biological properties or activities, known as the similarity-property principle (SPP). Molecular representations, such as chemical descriptors, can be extracted from the molecular structure and correlated through mathematical relationships with experimental values or biological activities of these molecules. QSAR modelling has grown in tandem with ML and provides valuable tools for drug development and the evaluation of different types of toxicity and adverse effects.

Conventional QSAR is typically characterised through linear regression explaining biological activity for a set of molecules. A QSAR model is able to predict changes in bioactivity as a function of structural modifications [105]. The evolution of QSAR modelling from linear models to more sophisticated ML models has already been discussed [106].

It is important to remark that QSAR models are not always applicable. This depends not only on its precision but also on its applicability domain; for example, structural modifications that lead to substantial bioactivity changes could be inconsistent with the SPP, remaining out of the applicability domain of linear QSAR models. The generation of a complete dataset that adequately matches the prediction of the target property represents a significant limiting factor for QSAR modelling, often far beyond the inherent capabilities of the technique. ( [107]).

Over the last few decades, a large number of publications have emerged applying different ML techniques for QSAR modelling. RF has been a popular choice, as it can perform excellent predictions using only a few parameters and is amenable of parallelisation. The current trend is towards DNNs. One of the first models based on feed-forward NNs was [108]. On the other hand, to overcome the disadvantages of unbalanced datasets, CNNs exploit hidden structures in the data to achieve better results [109]. Furthermore, it is interesting to highlight Karpov’s work as a pioneer in applying a transformer-based model to the QSAR task [110].

The application of QSAR modelling in medicinal chemistry projects has been extensive in the last decades, helping to solve problems in a wide variety of topics. One of its major applications has been the search for active compounds against a therapeutic target, where the QSAR model itself can be applied as a filter in the screening of chemical libraries [111–116]. Another important application is the development of models capable of predicting a relationship between chemical structures and different types of toxicity, such as In vitro toxicity [117], In vivo toxicity [118], mutagenesis [119] or hepatotoxicity [120], among others.

### Molecular docking

Molecular docking techniques are used to study the ligand–target interaction applying molecular mechanics to solve molecular interactions, being a key method in drug discovery and drug development [121, 122]. The docking process has two main stages: conformational sampling and prediction of the ligand position and orientation within the binding site; and binding affinity estimation.

Several algorithms have been developed to facilitate conformational sampling. The simplest one treats the ligand and the macromolecule as two rigid bodies, thus reducing to six the degrees of freedom. Another algorithm is incremental construction (IC), wherein conformational sampling is allowed by the fragmentation of the ligand from rotatable bonds into various segments. These two types of algorithms are fast, but their accuracy is somewhat lacking. Monte Carlo (MC) techniques allow for better sampling than the previous algorithms. With it, a ligand gradually modifies the bond rotation and translation or the position of the entire ligand. A major concern with the MC approach is the uncertainty concerning convergence, which can be better assessed through multiple independent runs. A related approach employs genetic algorithms (GA) to find global optima. GA retains a population of ligands with an individual suitability given by the scoring function (SF) and changes the ligands in the population through mutations or crossovers. The major limitation of GAs is their uncertain convergence. Finally, another interesting approach is the hierarchical method. By calculating low-energy conformations and their alignment, this method merges the ligand conformations into a hierarchy by clustering the most similar conformers. Subsequently, when performing ligand rotation or translation, the docking program will use this hierarchical data structure and thus minimise the results [123].

One of the critical elements in molecular docking is its SF. This one is needed to calculate an estimation of the binding affinity of protein–ligand complexes. A robust SF has to perform well in four tasks: scoring (obtaining binding scores); ranking (correctly classifying known ligands by their binding affinities); docking (identifying the native ligand pose); screening (selecting the true ligands among a random set of molecules) [124]. SFs are classified into two groups [125, 126]: classical and ML based. Classical SFs establish the relationship between the features that characterise a protein–ligand complex and its binding affinity and are classified as: force-field-based, empirical and knowledge-based. There is a wide variety of softwares that use these SFs robustly, especially for docking and screening purposes. However, these programs have room for improvement in scoring and classification tasks.

ML SFs are based on learning the correlation of the binding affinity of protein–ligand complexes and the features mapping the system through an ML algorithm. ML SFs beat classical ones systematically in all four tasks, especially in scoring and classification. Moreover, one of the competitive advantages that ML SFs have over classical ones is that they can better handle vast volumes of structural data. We outline some examples.

*Random Forest SFs*. RF-Score was arguably the first ML method achieving high performance in scoring terms [127]. It was developed using a RF algorithm and atom-type pair counts as characteristic features in order to describe the molecular complexes. Another example is SFCscoreRF [128] which is a SF based on SFCscore, an empirical scoring function [129], and derived with a RF from a large training set of target–ligand complexes extracted from PDBbind. Finally, one of the latest developments is VinaRF [130]. This SF applies RF to parameterise corrections to the AutoDock Vina SF [131].

*Support Vector Machines SFs*. ID-Score selected a set of 50 descriptors in order to describe protein–ligand interactions (covering nine categories of molecular interactions) and a SVM model [132]. More than 2200 complexes were used as the training set, and a modified support vector regression (SVR) algorithm was a benchmark test set, showing a considerable performance against other commonly used SFs.

*Artificial Neural Networks SFs*. The first work that confirmed that NNs can produce effective SFs was NNScore2.0 [133]. This SF was compared to Vina and AutoDock offering better performance, using two different metrics for docking efficacy (outputs of Vina and the BINANA [134] algorithm that provides 12 distinct ligand-target binding characteristics). The advance in the development of NN techniques towards DNNs, and particularly CNNs, has favoured the appearance of a large number of works in recent years in this field. One example is AtomNet, a deep CNN for the prediction of bioactive small molecules [135]. The accuracy of this CNN was evaluated on the Database of Useful Decoys-Enhanced (DUD-E) benchmark platform against previous SFs. Multilayer CNN models are able to successfully learn and differentiate between correct and incorrect binding poses when trained on 3D drug-target complexes using DUD-E [136]. CNN-based SF had a significantly better accuracy than AutoDock Vina in predicting both binding poses and affinities. DeepSite is a deep CNN that processes structural data as 3D images [104]; the CNN was applied to identify ligand binding sites showing better performance than the state of the art. A subsequent work developed KDEEP, a 3D graph CNN model that is able to predict ligand-protein binding affinities [137]. Such representation has the potential of enabling efficient pocket similarity search, pocket classification, and can serve as input for downstream ML algorithms. The Graph-CNN framework performed comparably well in predicting protein–ligand interactions with respect to other structure-based techniques without dependence on target–ligand complexes and demonstrated superior performance over ligand-based methods in difficult cases where there are small differences between active and inactive compounds [138]. Extended connectivity interaction features (ECIF) constitute novel descriptors of protein–ligand complexes; ECIF consists of 1540 possible protein–ligand atom pairs, which are simple to calculate and take into account atomic connectivity. Its underlying principle, however, is a flexible concept that could be applied to different types of complexes [139].

### Molecular dynamics simulation

The study of complex biological systems had a major boost due to the development of molecular dynamics (MD) simulations [140]. They facilitate the study of particle motion in a biological system applying classical mechanics, solving Newton’s motion equations. Physical interactions between particles can be described using quantum mechanics (QM), molecular mechanics (MM) or a mixture of both. The way in which the interactions between particles are described is critical to the accuracy of the simulations, as is the size of the systems to be simulated and the timescale that can be achieved. ML approaches can either take advantage of the information in MD simulations for the prediction of physicochemical properties, enhance the precision of force fields or learn the generation of equilibrium samples more efficiently.

ML has become a powerful tool for the development of high-precision force fields for MD. Some of the latest work has focused on new methodologies for the development of more accurate and efficient ML-based atomistic force fields for MD simulation [141, 142]. This kind of methodology interpolates between known training data, which were previously calculated *ab initio*, and the prediction of the target property, which are the new force field parameters. The generation of force fields is significantly simplified compared to classical force fields, which need manual adjustment of the parameters. Nevertheless, there is still the problem of how to choose proper training data.

A very efficient automatic way to solve such problem is through on-the-fly ML methods [143]. On-the-fly ML could equally be viewed as a First Principle Molecular Dynamics (FPMD) approach, where the needed QM information is only computed to increase the database at each step of the simulation while retaining the vast applicability of FPMD. During the run of MD calculations, *ab initio* data are selected and added to the training data. As long as the dynamic visit configuration is well represented in the existing database, no additional QM calculations should be performed. This should only happen if a new event requires it, and this is how the on-the-fly ML workload is minimised.

On-the-fly ML permanently generates a force field from the existing data generated in the MD simulation. This technique is based on direct ML prediction of atomic forces. These are estimated through Bayesian inference using standard GP regression. This requires constructing a required covariance matrix. For this purpose, an efficient representation is needed to describe atomic configurations and a function calculating the distance between any two such configurations suitable in order to predict the atomic force. This technique has been applied in several recent works, demonstrating that it can improve the results obtained with conventional approaches [144–146].

The crucial element for on-the-fly ML is the probability model for error estimation. At each step, a decision is made whether to do an *ab initio* calculation and possibly add the data to the force field or use this one for that step and omit learning for that step (hence the “on-the-fly” learning name). Thus, the more accurate the force field, the less sampling is needed, and the more costly *ab initio* steps are omitted. In this way, the convergence of the force field can be controlled, but an extensive scan through the phase space for training structures can be performed.

ML is also implemented in MD to accelerate and optimise the trajectory production. In particular, this can be done *a posteriori*, i.e. by running a biased sampling of the collective variables (CVs) once identified from the analysis of one or more exploratory MD simulations (MD/ML resampling), or through on-the-fly protocols. On-the-fly learning represents an elegant way to combine MD with ML. In particular, they share with adaptive sampling procedures, the feature of taking care of launching and controlling repeated sequences of multiple MD simulations in an automated manner. ML methods help in optimally identifying the starting states for each series of MD runs. In practice, a broader exploration is achieved without the need of introducing external biases [147].

ML has also been successfully used to analyse longtime scale simulation data in large systems [148], for predicting ground state energies [149], molecular atomisation energies [150] or even predict the solvent-solute interaction map [151] demonstrating the versatility and applicability of ML in improving MD techniques.

## Further issues

We have provided a brief review of potential and actual uses of AI and ML methods in drug development with emphasis on molecular modelling applications. However, there are many other relevant applications in other areas which we sketch here.

Let us mention first the *identification of molecular targets to find hit or lead compounds*. The deluge of molecular biology data together with the evolution of AI has allowed the transformation of molecular target identification methods. As an example, the SPiDER software uses self-organising maps to predict drug equivalence relationships [152]. Numerous other techniques including SVMs, RFs and NNs have been used for virtual screening of hit compounds [153]; similarly, AI techniques have proved useful in predicting the ensuing best synthetic routes [154, 155] or enantiomeric excess [156].

Concerning *preclinical studies*, recall that drug safety and toxicity issues, both during development and postmarketing, constitute a major challenges for the industry, mainly due to the difficulty of correlating animal and human data [157]. AI can promote safer drugs through the use of ML models at preclinical stages to deal with data obtained from adverse event monitoring of drugs [158]. Several platforms like DeepTox [159] and PrOCTOR [160] are available. Finally, *clinical trials* constitute another bottleneck in drug development, taking about half the time and cost of getting a drug to market; several ML- and DL-based tools are helping in clinical trial design [6, 7].

Interpretability and explainability are emerging as major issues when applying ML in drug development. As an example, the acceptance of a QSAR model depends not only on its accuracy but also on its interpretability. In that sense, the parameters in deep models are often abstract and disconnected from the real world, which complicates result explanations. When properly trained, predictions obtained by NNs may have a high accuracy. However, MC often perceives them as black boxes, their insights remaining mostly opaque. There are various approaches to the problem as thoroughly reviewed in [161]. One possibility is to use interpretable models, easily comprehensible for humans, as cogently argued by [162] who claims that in many contexts we may perform with such models almost as well as with deep ones.

Another relevant ML issue in MC, briefly mentioned above in relation with ChemBERTa, is transfer learning [163, 164]. The training of huge neural models requires large amounts of labelled data, typically in the order of thousands to millions. In cases where human labelling of training data is not feasible with those magnitudes, it is possible to leverage similar datasets, even not for the same task. It fundamentally entails adopting a model previously trained over a massive dataset and then fine-tunes it in the final task, with a much smaller dataset. The adoption of pretrained models allows the practitioner to save in computational costs, often leading to good enough performance. In addition, the quantity of labelled data can be drastically reduced by strategically choosing the data points to be annotated. Techniques developed to automatise this idea fall under the term of active learning, and the Bayesian approach offers a principled and sound framework for it, e.g. [165].

## Challenges

We end up the paper describing several challenges concerning the use of AI in drug development.

We have mentioned several advantages that Bayesian ML methods may have in this area. In particular we would stress their ability to be integrated coherently within a decision-making structure. After all MC end up making numerous decisions during the process. The methods here described support prediction, as frequently showcased, but we feel there should be further emphasis in the decision support aspects [17].

There are also several important technical challenges. Most importantly, efficient Bayesian integration methods in DNNs are still to be found. In particular their solution would facilitate the development of probabilistic programming languages [166, 167]. This would lead to new tools that would facilitate the democratisation of these techniques to the MC community at large.

Another important challenge in the medicinal chemistry area refers to the acquisition of sufficient and high-quality data in order to be able to develop robust models. It would be of great help if the information were shared; however, this is a difficult obstacle to overcome, due to data confidentiality. Researchers are already working to find a solution to this important limitation. As an example, Altae-Tran et al have developed an algorithm based on *one-shot learning* that can be used to significantly lower the amount of data required [168].

Getting good molecular representations of structures is yet another important challenge that remains to be solved. Recent theoretical models learn task-related features from raw data and then refine the molecular representation to a standard [169]. Another important challenge is to achieve accurate predictions of binding affinity between a target protein and a drug.

In the meantime, further multidisciplinary integration between MC and ML researchers would certainly benefit both fields.
